# Hydrothermal liquefaction of sewage sludge anaerobic digestate for bio-oil production: Screening the effects of temperature, residence time and KOH catalyst

**DOI:** 10.1177/0734242X221138497

**Published:** 2022-11-19

**Authors:** Stian Hersvik Hegdahl, Camilla Løhre, Tanja Barth

**Affiliations:** Department of Chemistry, University of Bergen, Bergen, Norway

**Keywords:** Bio-oil, hydrothermal liquefaction, sewage sludge, biogas, digested sewage sludge

## Abstract

Due to sewage sludge being an abundant biobased resource, and with the number of biogas plants utilizing sewage sludge increasing, digested sewage sludge (DSS) is a promising feedstock for producing bio-oil. This study uses DSS from a biogas plant to produce bio-oil in a hydrothermal liquefaction process adjusting time from 2 to 6 hours, temperature from 280 to 380°C and the presence of a base as a depolymerization agent and potential catalyst. High conversion yields are obtained, with the maximum of 58 wt% on a dry, ash free basis and an energy recovery of up to 94%. The oils contain compounds with a potential for utilization as biofuels and building blocks, especially fatty acids as biodiesel feedstock and biobased phenols, glycols and aliphatic alcohols.

## Introduction

In a circular economy perspective, the identification of waste resources that can be used as feedstocks for new products is a critical factor. Municipal wastewater sludge from sewage is one such resource, and anaerobic digestion of the sludge is a well-established technology for energy recovery as biogas (Seiple et al., 2020). The number of biogas plants is increasing, and several countries have ambitions of producing and utilizing large amounts of biogas in the near future (European Biogas Association, 2018). This provides promising perspectives for use of sewage sludge as a biogas feedstock. In 2020, Bergen Biogas Facility produced approximately 14.8 × 10^6^ Nm^3^ of upgraded methane, providing an energy supply of 53.2 TJ. This was based on 5720 tonnes of sludge on a dry basis (personal communication, 2021). After the biogas is produced, however, the facilities are left with a residue of digested sewage sludge (DSS) which must also be disposed of. As of today, the DSS is exported out of the area and used for soil improvement elsewhere.

As sewage sludge is an abundant feedstock, and as biogas plants are continuously being built, more DSS will be produced over time. Therefore, DSS is a feedstock that is attracting increased research focus (Peccia and Westerhoff, 2015; Prestigiacomo et al., 2019; Tsybina and Wuensch, 2018; Vardon et al., 2011). One use for the DSS is as a fertilizer and soil conditioner. This is frequently limited by a high content of heavy metals, and also in some cases by an excess of phosphorus, potentially leading to metal contamination and eutrophication (Hordaland Fylkeskommune, 2017). In such cases, fertilizer application of DSS often leads to a requirement for large areas of arable lands to utilize the bioresidue, and the resulting transport is both expensive, inconvenient and environmentally challenging. In addition, the DSS contains significant amounts of water, which will thus increase the volume and add to the transport costs. Such considerations are the basis for evaluating the alternative use of DSS as a feedstock for thermochemical processing, potentially combined with nitrogen and phosphorous recovery. In recent life cycle and techno-economic analyses, hydrothermal liquefaction or carbonization (HTL or HTC) are evaluated to be the most promising technologies for retrofitting of existing municipal treatment facilities already producing biogas (Mayer et al., 2021; Tian et al., 2020).

HTL and HTC are preferred technologies for thermochemical conversion of wet feedstocks, as drying is not required. Comparing liquefaction (HTL) to carbonization (HTC), the liquid bio-oil has a wider applicability than the solid carbon-containing product from HTC, since as the bio-oil can be upgraded and used as a renewable motor fuel component and a source of biobased chemicals. We therefor consider HTL the most attractive conversion technology, as also suggested in the review by Angenent et al. (2018).

Research addressing HTL processes, where the biopolymers in biomass are depolymerized into bio-oil in a liquid reaction medium at elevated temperature and pressure over time, has over the last decades been given considerable attention (Brown and Stevens, 2011; Castello et al., 2018; Clark and Deswarte, 2008; Dimitriadis and Bezergianni, 2017; Gollakota et al., 2018). In 2008, a variation to this process was developed using formic acid (FA) as an in situ hydrogen donor. This process was called the lignin-to-liquid (LtL) process as this study was done on lignin-based feedstocks (Kleinert and Barth, 2008). The LtL process has been continuously developed since then, including the use of water as the reaction media, as described by Løhre et al. (2016). Other additives can also give increased yields, and in 1999 a study was done showing positive effects of adding a base to woody biomass HTL conversion, where the base functions as a depolymerization agent and potential catalyst. The study found best results with added sodium hydroxide (Borgund and Barth, 1999); however, subsequent unpublished findings have provided even better results by adding potassium hydroxide. This is also found in a more recent paper from 2015 by Nazari et al. (2015).

However, these investigations and optimization studies mostly use lignocellulosic biomass or their lignin fractions as feedstocks, while conversion of biosolids from sewage sludge and residues from biogas production have been much less explored. These feedstocks have a highly variable composition, depending on the local municipal solutions and the microbial ecology at the fermentation stage (Jiang et al., 2021). Though some lignocellulosic biomass is present, and especially non-digestible lignin fractions, a large fraction of the organic matter will consist of microbial biomass. This increases the content of organic nitrogen in the DSS and makes it to some degree more comparable to micro-algae-based feedstocks than lignocellulosic biomass. The level of inorganic compounds is also considerable and will vary with the local conditions and may catalyze or inhibit some thermochemical reactions.

Of the papers focusing on HTL with sewage sludge and DSS as feedstocks, reports on the results from DSS are quite few, as presented in a recent review paper by Cheng et al. (2020). Some relevant examples are found: Vardon et al. report bio-oil yields of 9.4% (300°C for 30 minutes) (Vardon et al., 2011) and Marrone et al. present oil yields from digested solids of 34.4 wt%, dry, ash free (*daf*) with a H/C ratio of 1.4 (332–358°C, continuous flow with a residence time of 30 minutes) (Marrone et al., 2018). Trabelsi et al. have performed a centred composite experimental design for pyrolysis of municipal sewage sludge. This study gave a bio-oil yield of up to 28.0 wt% (Trabelsi et al., 2021).

Castello et al. have reviewed the use of continuous HTL for biomass and waste feedstocks. The review includes a graph showing the energy recovery relative to the oil yield. These studies are based on different feedstocks, including sewage sludge, algae and wood (Castello et al., 2018). Prestigiacomo et al. report energy recoveries well above 100%, suggesting that energy is stored in the product compounds (Prestigiacomo et al., 2020). Fan et al. have specifically addressed the reactions of N-containing constituents in HTL using model compounds (Fan et al., 2020), which are relevant for molecular analysis of oils from feedstocks containing microbial biomass.

The chemical complexity of the DSS makes a theoretical estimate of optimal conversion parameters impossible. A pragmatic approach using experimental design and optimization is a much more viable alternative. A few optimization studies on thermochemical conversion of biosolids from sewage sludge has previously been reported: HTL of SS optimization is described (Madsen and Glasius, 2019), and Trabelsi et al. (2021) used a central composite design for their optimization of fast pyrolysis of SS. However, the different composition of the DSS compared to raw SS, and the inherent differences in sewage sludge composition with regard to treatment plants, technologies, seasons and other variable parameters, make optimization of each type of SS derived feedstock necessary.

In this work, an experimental design is used for optimizing HTL of DSS from the Bergen municipal biogas plant. The HTL conversion performed with addition of FA as a hydrogen source as used in established protocols (Ghoreishi et al., 2019; Løhre et al., 2016). The process variables that are tested comprise reaction temperature, residence time and addition of KOH (catalyst). The molecular composition of the produced oil and elemental analysis of oil and residual solids is analysed and provides a basis for mass recovery calculations and for calculation of the energy recovery in the bio-oil. Thus, this study provides a complete data set for evaluation of the use of HTL as a final valorization step in DSS processing, including the provision of optimal HTL conditions to maximize bio-oil yields from DSS.

## Material and methods

### Conversion of DSS to bio-oil

The DSS used in this study was provided by Bergen Biogas Facility (Norway), after anaerobic digestion of the sewage sludge was completed. The input for biogas production is supplied by wastewater treatment plants around Bergen (Akervold 2016). The feedstock was dewatered at the Bergen Biogas Facility.

Non-dried DSS (4 g), FA (1 cm^3^) and distilled water or KOH (1 cm^3^, 1 M) were added to a nonstirred 25 cm^3^ 4740-series Parr reactor (Parr Instrument Company, Moline, Il. USA). The reactor was closed and heated at 280–380°C in a preheated Carbolite Laboratory High Temperature oven for 2–6 hours. After heating, the reactor was removed from the oven and cooled to room temperature. The gaseous phase was vented and the contents of the reactor were filtered to gain the solid phase. This was done first by filter paper (Ahlstrom-Munksjö, Helsinki, Finland), secondly by glass fibre filter (Whatman, GF/A, Whatman plc, Maidstone, Kent, UK), using ethyl acetate (EtOAc) and tetrahydrofurane (THF) (9:1, v/v) as a solvent. The aqueous phase was separated from the organic phase by decanting before the organic phase was dried over sodium sulphate, and evaporated on a rotary evaporator at 40°C, 200 mbar. The gaseous phase was determined by weighing the reactor before and after venting the gas. The solids were determined by weighing the equipment used before and after filtration and drying. A portion of the solids was dried in a heating cabinet at 70°C for 3 days to determine the dry matter content. The oil yield was determined by mass after solvent evaporation. Water recovery was not measured due to a significant loss of water during evaporation of EtOAc.

### Experimental design

The experimental conditions were set up as a full factorial design (Carlson, 1992: 89–100; 123–135), where the three varying factors were temperature (280 or 380°C as low and high values), time (2 or 6 hours as low and high values) and the presence of KOH as a depolymerization agent and potential catalyst. In the case of KOH addition, 1 cm^3^ of 1 M KOH was added, while in the cases of no KOH addition, 1 cm^3^ of water was added to keep the filling constant. Two experiments were performed with temperature and time at centre values (330°C and 4 hours), both with and without KOH. All experimental conditions are given in Table 2.

### Design and multivariate data analysis

To study the results from the experimental design, principal component analysis (PCA) was performed using Sirius 11.0 to produce a biplot. A biplot shows positively correlating factors on the same side of the origin (shown by a plus sign in the results), while negatively correlating factors are on opposite sides of the origin. Factors that are at 90° angles from each other through the origin, are not correlated. The larger degree a factor affects or is affected by other factors, the further away it is from the origin (Carlson, 1992: 342–351).

In addition to the PCA, a partial least squares (PLS) method was used, as implemented in Sirius 11.0, which quantifies the effect each factor has on the chosen result (Carlson, 1992: 459–469). Factors considered insignificant to each result were removed from the equation. This was also controlled by considering the change of the explained factor and *R*-value after removal of the factors.

### Gas chromatography coupled with mass spectrometry

All oils were analysed by GC-MS after silylation. The instrument used was an Agilent Technologies (Santa Clara, CA, USA) 7890 A GC with auto-sampler. The detector was an Agilent 5977A MSD. Injection was done in splitless mode with an injection temperature of 280°C. The column from Agilent Technologies was a 30 m HP-5ms with a thickness of 0.25 μm and Internal diameter (ID) of 250 μm. The temperature program was set as follows: 50°C 2 min^−1^, 10°C min^−1^ to 200°C, 20°C min^−1^ to 300°C 5 min^−1^.

For the mass spectrometer detector, the ion-source temperature was set to 230°C with a mass range from 25 to 400 μ, with a positive ionization at 70 eV. The solvent delay was 5.50. The software used for identification of the compounds was Enhanced Data Analysis, MSD ChemStation, coupled with the NIST 2.0 library.

### Silylation of the produced oils

To improve GC separation, silylation was done by adding the oil (0.01 g) to a vial. This was dissolved in EtOAc/THF (3.000 cm^3^, 9/1, v/v) containing hexadecane as internal standard (0.01 mg cm^−3^); 1.000 cm^3^ was taken for further use before pyridine (0.150 cm^3^) and N,O-bis(trimethylsilyl)trifluoroacetamide (0.150 cm^3^) were added to the mixture. The vials were capped and heated at 70°C for 30 minutes before cooling to room temperature. The silylated bio-oil (0.700 cm^3^) was transferred to a new vial, mixed with pentane (0.700 cm^3^), resulting in a solution containing approximately 1.3 mg cm^−3^ of the silylated oil, and 4.2 μg cm^−3^ of the internal standard. The samples were cooled at 5°C overnight before filtration through a 0.45 μm Puradisc NYL filter prior to GC-MS analysis.

### Elemental analysis

Two parallels of all oils were analysed in CHNS mode with a Vario EL III instrument using He as carrier gas. The instrument used is not calibrated to measure sulphur. The amount of oxygen was calculated by difference.

## Results and discussion

### Feedstock

The DSS feedstock, provided by Bergen Biogas Facility, was reported to be 28.9 wt% dry matter, whereof 58.1 wt% was organic material. Information on the bulk composition of the feedstock is found in Table 1. The inorganic constituents of the feedstock include iron, aluminium, phosphorus, nitrogen (ammonia and nitrates) as well as other heavy metals (analysis by the Biogas Facility). The values are specific for the batch used in the experiments within this study as the composition will vary between batches.

**Table 1.** Feedstock information.

Feedstock	Digested sewage sludge
Dry solid content (wt%)	28.9
Ash_dry_ (wt%)	41.9
Dry, ash free content (wt%)	16.8
C_dry_ (wt%)	28.2
H_dry_ (wt%)	4.5
N_dry_ (wt%)	3.1
O_dry_ (wt%)^a^	22.3
H/C_molar ratio_	1.9
O/C_molar ratio_	0.6
N/C_molar ratio_	0.1
HHV (MJ × kg^−1^)^b^	11.9

a Oxygen content was found by difference.

b Higher heating value (HHV) is found by the formula presented by Channiwala and Parikh (2002) and is based on dried feedstock.

### Experimental design

The HTL screening was performed as batch experiments at laboratory scale, as described in the experimental section. The DSS was used as supplied, with 28.7% dry content in water, with an addition of FA (1 cm^3^ per 4 g DSS) and KOH or water (1 cm^3^ 1.0 M KOH per 4 g DSS). The screening was set up as a full factorial design (Carlson, 1992: 89–100; 123–135), where the three variable factors were reaction temperature, residence time and the presence or absence of KOH as a depolymerization agent and potential catalyst. The values of the reaction temperature and residence time were based on previous screening of lignocellulosic feedstocks (Ghoreishi et al., 2019; Løhre et al., 2016) and were chosen to cover a wide range of oil conversion. Replicate experiments were performed to assess the reproducibility. All experimental conditions are given in Table 2.

**Table 2.** Experimental conditions.

*T* (°C)	*t* (hours)	H_2_O/KOH	Conditions^a^	Naming of experiment^b^
280	2	H_2_O	− − −	280.2.H_2_O
380	2	H_2_O	+ − −	380.2.H_2_O
280	6	H_2_O	− + −	280.6.H_2_O
380	6	H_2_O	+ + −	380.6.H_2_O
280	2	KOH	− − +	280.2.KOH
380	2	KOH	+ − +	380.2.KOH
280	6	KOH	− + +	280.6.KOH
380	6	KOH	+ + +	380.6.KOH
330	4	H_2_O	0 0 −	330.4.H_2_O
330	4	KOH	0 0 +	330.4.KOH
280	6	KOH	− + +	280.6.KOH_2
380	6	H_2_O	+ + −	380.6.H_2_O_2
380	6	H_2_O	+ + −	380.6.H_2_O_3
380	6	KOH	+ + +	380.6.KOH_2

a The +, 0 or − notation indicate whether the factors are in their high, centre point or low values.

b The experiments are coded by HTL temperature, residence time in hours and whether 1 M KOH or water was added.

### Bulk yields

**Figure 1.**
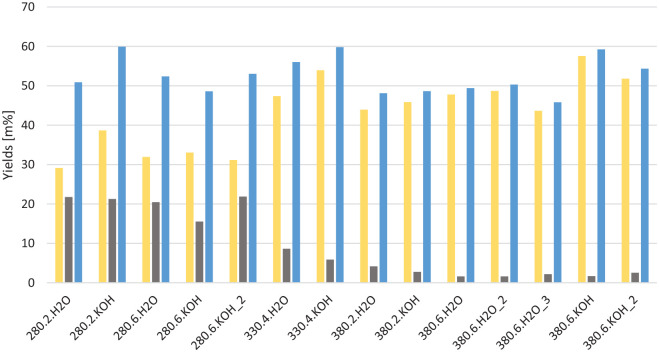
Yields of oil (yellow), organic fraction of the coke (grey) and total oil and coke yields (blue) for each experiment, on dry, ash free basis, given as % mass relative to organic input. The experiments are coded by HTL temperature, residence time in hours and whether 1 M KOH or water was added. HTL: hydrothermal liquefaction.

The yields of oil and coke/solids are shown in Figure 1 on *daf* mass basis. This also includes two experiments performed in duplicates, and one experiment performed in triplicate for comparison of the results. These are all shown in Figure 1 and indicate uncertainties of 1–3 wt% of the oil yields. The oil yields are in the range of 29–58 wt%, *daf*, and there is a clear trend showing that increasing temperature increases the oil yield. The solid phase ranges from 39 to 74 wt%. The solids have an organic matter content of 2–22 wt%, which confirms that the major part of the organic products are found in the oil phase. The total mass recovered as oil and solid organic matter is in the 45–60 wt% range relative to the input, which shows that water, gaseous products and dissolved organic compounds are also produced in significant amounts.

### Elemental composition and carbon and energy recovery

**Figure 2.**
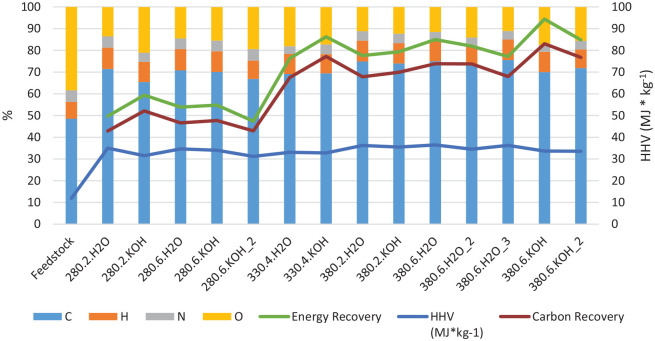
Elemental composition of the bio-oils and DSS feedstock, together with the carbon and energy recovery and the estimated heating value (Channiwala and Parikh, 2002) of the bio-oil. The experiments are coded by HTL temperature, residence time in hours and whether 1 M KOH or water was added. HTL: hydrothermal liquefaction; DSS: digested sewage sludge.

Since the HTL conversion involves significant restructuring of the molecular composition of the constituents in the oil compared to the initial biomass, yield calculations on a mass basis are not optimal for evaluating the efficiency of the conversion. The energy and carbon recovery in the oils and the heating value of the product are much more relevant. Figure 2 shows the elemental composition of the bio-oil and the carbon recovery (found by the mass of carbon in the oil divided by the mass of carbon in the feedstock, both of which are determined by elemental analysis) as well as the energy recovery (found by *m* × HHV of the oil divided by *m* × HHV of the dry feedstock). This is also listed in Table S1 in the supplemental material. FA is not included in the energy recovery calculations, as previous work has shown only minor incorporation of FA degradation products into the bio-oils (Halleraker et al., 2020).

Based on the HHV values for the oils (shown in Figure 2 and given in Table S1 in the supplemental material) as well as the oil yields, a rough estimate is possible to do on the energy received from combustion of the bio-oil. While Bergen Biogas Facility produced biogas comprising 53 TJ in 2020 (values received from Bergen Biogas Facility), an estimate of hydrothermally treating the bioresidue gives an additional 1000 tonnes bio-oil with a total energy content of 34 TJ.

For the energy recovery in the bio-oil relative to the DSS feedstock, the maximum is in the range 85–95% at the highest temperature and longest residence time. This is a very high value, especially since the energy remaining in the gas, solid and dissolved products are not included due to lack of detailed analysis of the aqueous and gas phase. For comparison, Prestigiacomo et al. reported overall energy recovery values from up to 150% of the initial chemical energy in undigested sewage sludge solids after HTL conversion at 350–400°C, with around 70% of the energy contained in the bio-oil (Prestigiacomo et al., 2020). The energy recovery reported for their bio-oil is thus lower than the results reported here. Since we lack quantification and elemental analysis on the additional phases, a complete energy balance is not possible, but it is reasonable to expect it to pass 100% within our system as well. This would mean that thermal and chemical energy is stored in the bio-oil due to endothermic reactions, typically with incorporation of hydrogen from the FA and water in the reaction medium. However, such hydrogen incorporation is not obvious from the elemental ratios, and thus the basis for the high energy recovery still needs to be clarified.

The maximum carbon recovery of 83% of the input carbon found in the oil phase is observed at the highest temperature and longest residence time with KOH present (experiment 380.6.KOH). However, its replicate experiment (experiment 380.6.KOH_2) gives only 76.7% C recovery in the oil, so there is some variability in the results. High carbon recovery values are also found at the same conditions without KOH addition, 73.7 and 73.9%. The estimated energy recovery depends both on the elemental composition of the bio-oil and the quantitative yield, and also reaches a maximum of 94.4% for the 380.6.KOH experiment.

**Figure 3.**
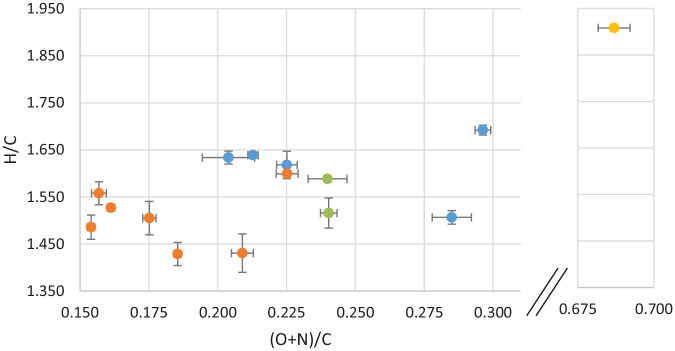
van Krevelen diagram of average bio-oil values coded by temperature, with error bars, where blue is low temperature experiments (280°C), green is middle temperature experiments (340°C), orange is high temperature experiments and the feedstock is shown in yellow.

HTL in the presence of FA is expected to increase the hydrogen content in the oils, since the FA decomposes at these temperatures and can provide hydrogen radicals (Kleinert and Barth, 2008). In the experiments reported here, the hydrogen content in the oil is in the 8–10% range. However, the DSS initially had a higher H/C ratio than the bio-oil products, as shown in Figure 3. The major change in the elemental ratios is the reduction of the oxygen content. This suggests that elimination of water from DSS carbohydrate constituents can be a significant reaction pathway.

In the van Krevelen diagram given in Figure 3, the oils are coded by temperature. In the diagram, the average of two parallel analyses from a single experiment is shown, including the uncertainty. Duplicate measurement of samples from the same experiment generally show much closer values than those from parallel experiments, which again suggests some variability in the feedstock. The HTL conversion more than halves the (O+N)/C, but also slightly decreases the H/C. As for the oils themselves, the figure shows the oils produced at high temperature (380°C, shown in orange) to have a lower H/C and (O+N)/C than the oils produced at low temperature (280°C, shown in blue). This trend is supported by the middle-temperature oils (330°C, shown in green), positioned in-between the high and low temperatures. However, the range of variation in the H/C values are not great.

The maximum oil yield obtained was 57.6 wt%, *daf*, with a H/C = 1.6 and an energy recovery of 94%. This result was received from the experiment with high values of all experimental variables (380.6.KOH). Previously published findings for a similar DSS feedstock show a yield of 34.4 wt%, *daf* with a H/C of 1.4 in a continuous flow HTL-system run at 332–358°C, 20 Mpa pressure and 30 minutes average residence time (Marrone et al., 2018). The maximum yields reported here are thus much higher, showing the importance of optimizing the conversion conditions.

N/C ratios in the oils are halved relative to the DSS starting material (shown in Table S1 in the supplemental material). This suggests that water soluble nitrogen-containing compounds are produced in considerable amounts. Further analysis of the water phase will be performed and presented at a later stage.

### Bio-oil composition analysed by GC-MS

**Figure 4.**
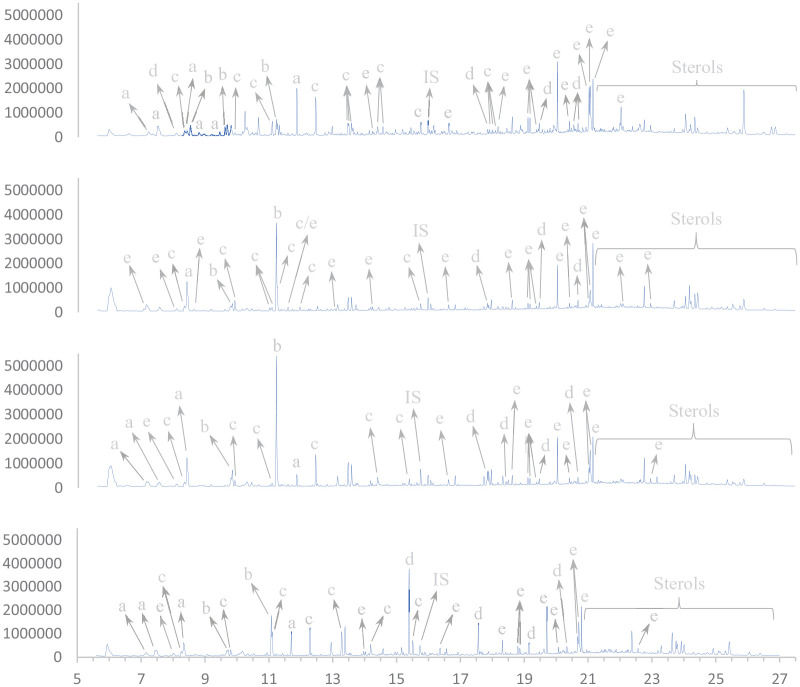
Compound classifications from selected oils. From top to bottom: 280.2.H_2_O; 380.6.KOH; 330.4.H_2_O; 330.4.KOH. a, glycols; b, hydroxycarboxylic acids; c, phenols; d, aliphatic alcohols; e, fatty acids. IS: internal standard.

The bio-oils have been analysed by GC-MS and comprise a large number of compounds, as shown in Figure 4. There are some variations based on the conditions used to produce the oil, but the main components are fatty acids, aliphatic alcohols, glycols and hydroxycarboxylic acids, as well as aromatics and ring structures such as phenols, sterols, pyridines, pyrimidines, pyrazines and pyrrolidines. Some compounds, such as glycols and carboxylic acids, tend to have a higher relative concentration in the oils at low reaction temperature (280°C). Other compounds, such as aliphatic alcohols, tend to have a higher relative concentration in the oils at high reaction temperature (380°C)

A complete overview of the identified compounds is given in Table S2 in the supplemental material.

### Principal component analysis

Use of an experimental design in a screening approach, with simultaneous variation of several experimental factors in a statistically balanced manner, gives the opportunity of extracting much more information about the reaction systems than testing one variable at a time. This includes the possibility of detecting interactions between several experimental factors. However, the direct interpretation of the results is not as intuitive, and systematic data analysis approaches are needed. To discuss the effects of the system variables investigated in this set of screening experiments we use PCA and PLS (Carlson, 1992). Since we use a full factorial design, the importance of the two-factor interactions can be included in the evaluation, for example, the combined effects of temperature and residence time (*T* *×* *t*) or temperature and addition of KOH as catalyst (*T* × KOH).

**Figure 5.**
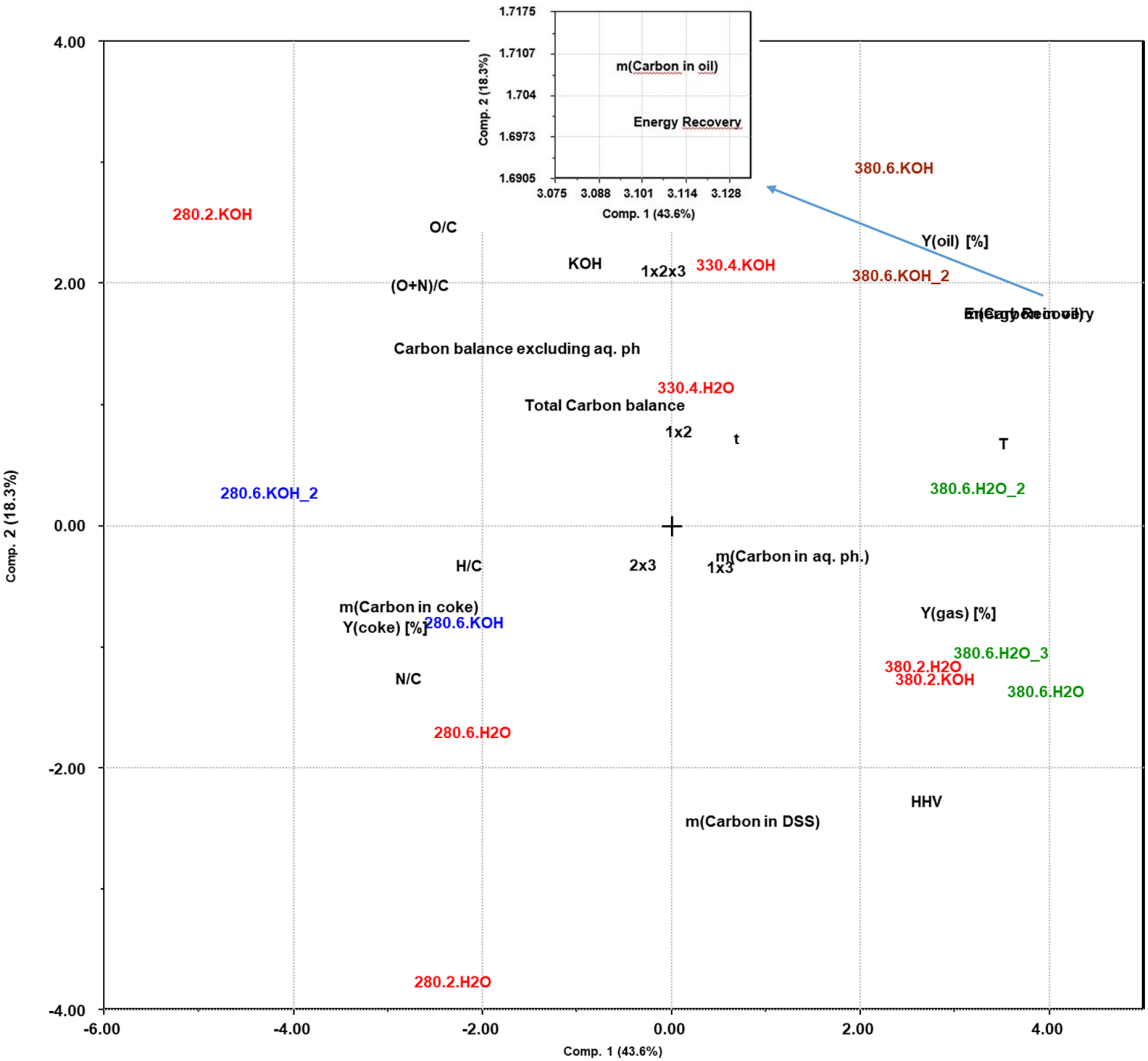
Principal component analysis, biplot showing relative correlations between reaction conditions and results.

As an initial overview, PCA is used to show how the yields and elemental composition of the oil (the responses) depend on the variable experimental factors (reaction temperature, residence time and presence/absence of KOH). Figure 5 shows a biplot of all the experiments where the correlation between experimental variables and the results can be observed. The first two components describe 61.9% of all the variation in the response variables, proving that there is a considerable degree of statistically significant variation in the overall data set. The biplot shows both the loadings of each experimental variable in defining the principal components (PCs) and how the outcomes of the experiments correlate with these factors. On the first component (PC1), which describes 43% of the variation in the data set, the temperature clearly is the experimental factor that has the highest impact. The oil yield, both on a weight basis and as carbon recovery, the energy recovery and the HHV of the oil, are all strongly positively correlated with the reaction temperature. The gas yield is also clearly temperature dependent, but since this is mainly a product of decomposition of the added FA and not a product from the DSS (Halleraker et al., 2020), it is of less relevance. The amount of carbon remaining in the coke is correspondingly negatively correlated to the temperature. This is probably a combined effect of the presence of unconverted biomass at low temperatures and increasing conversion of condensed carbon products/coke at the highest temperature.

On PC2, which describes 18.3% of the dataset variation, the presence/absence of KOH is the most significant experimental variable. It correlates positively to the oil yield (by weight) and the O/C ratio, and less strongly to the carbon recovery in the oil. The HHV of the oil correlates negatively to the KOH variable. Taken together, these observations suggest that one effect of adding potassium hydroxide is to retain oxygen in the bio-oil, increasing the mass but not the energy recovery (see Table S1) in the oil phase.

As for the elemental compositions, a negative correlation is seen from both temperature and time to the H/C ratio, while the KOH catalyst has a slight positive correlation. This positive correlation could possibly be considered insignificant, being close to 90° from H/C. The (O+N)/C is positively correlated to the KOH catalyst, while it is negatively correlated to the temperature. As the time factor is near the origin, as well as being close to 90° from ((O+N)/C, this is considered insignificant. The overall trends are that the heteroatoms O and N are removed from the bio-oil at the higher temperatures. In addition, some hydrogen is removed relative to the starting composition, possibly by dehydration reactions.

### PLS regression

The influence of the experimental factors on the experimentally measured yields can be described more precisely and quantitatively using a multivariate regression (PLS). Individual regression models describe the importance of each experimental variable on the selected experimental results, and the fit of the regression is evaluated as the regression coefficient *R*. Table 3 lists the equations for the PLS models for each experimental result. The equations are simplified to only include the variables that have a significant effect on the *R* value. In the equations, temperature (*T*) is the experimental variable that is found to have a high impact in all the regression equations. It is the only variable that systematically influences the overall energy recovery, where a simple linear regression line describes the relationship with *R* = 0.89. The temperature also correlates positively to the oil yield, energy recovery and HHV and negatively to the carbon content of the coke and the N/C and O/C ratios of in the oil. The residence time of the experiments (*t*) is only directly significant for the H/C ratio, but also appears as a cross factor with temperature (*T* *×* *t*) in several of the regression equations. The presence of KOH increases the O/C ratio but does not significantly affect the H/C or N/C ratios.

**Table 3.** Regression equations, showing the relationship between reaction conditions and results.

Factor	*c*	*T*	*t*	KOH	*T* *×* *t*	*T* *×* KOH	*t* *×* KOH	*T* *×* *t* *×* KOH	e.v. (%)	*R*
Y (oil)	−1.217	0.842	–	0.232	0.122	–	–	0.122	78.90	0.888
Y (coke)	7.950	−0.966	–	–	–	–	–	–	93.29	0.966
Y (gas)	17.75	0.522	–	−0.480	–	0.283	–	–	73.02	0.858
H/C	24.65	−0.617	−0.291	–	–	–	–	0.219	55.94	0.748
O/C	6.884	−0.414	–	0.415	0.183	–	–	0.339	67.57	0.822
N/C	10.83	−0.731	–	–	−0.348	–	–	–	75.49	0.871
(O+N)/C	9.093	−0.605	–	0.495	–	–	–	–	70.55	0.840
HHV	16.99	0.479	–	−0.560	−0.140	–	–	–	63.38	0.796
ER	−1.699	0.890	–	–	–	–	–	–	79.28	0.890

*c*: coefficient; e.v.: explained variance; *R*: coefficient of determination; ER: energy recovery.

## Conclusion

The experimental results show a maximum oil yield of 58 wt% of the organic fraction of the feedstock, which is higher than any previously reported value. The estimated energy recovery in the oil is higher than the weight-based oil value, reaching a maximum of 94% of the original energy content of the feedstock. This is primarily due to the elimination of oxygen from the oil components, reducing the O/C ratio from the initial value of 0.6 to a minimum value of 0.16 at the highest temperatures. Thus, the evaluation of the process efficiency needs to be done on a carbon basis and not just by weight. Overall, the results show that HTL conversion can increase the energy yields from SS residues by nearly 40%, while solving problems of deposition for the DSS.

Altogether, this set of screening experiments show that the preferred conditions for maximizing the oil yields and the energy recovered in the oil within the range of conditions tested is to use the highest temperature (380°C). The effect of adding KOH is small, mostly increasing the oxygen content in the oil, and the residence time of the HTL treatment is not important and can be adjusted to practical considerations.

The molecular analysis by GC-MS shows that the oil composition is dominated by oxygenated compounds like fatty acids, alcohols and phenols. Only minor amounts of hydrocarbons are found. Thus, the bio-oil composition is not directly suitable for motor fuel use but has a good potential for upgrading into useful products like biodiesel and biobased chemicals. The distribution of the compounds found in the oil differs based on the reaction conditions, where the temperature has the strongest influence on both the yields and composition. Potentially, a further increase in temperatures above the 380°C used here could still increase the yields and improve the oil quality. Overall, this set of screening experiments clearly demonstrate the high potential of DSS bioresidues as a sustainable waste feedstock for thermochemical production of bio-oils, and there is a very good potential for production of energy and biobased fossil replacement chemicals from DSS bioresidues subsequently to the initial biogas production.

## Supplemental Material

sj-docx-1-wmr-10.1177_0734242X221138497 – Supplemental material for Hydrothermal liquefaction of sewage sludge anaerobic digestate for bio-oil production: Screening the effects of temperature, residence time and KOH catalyst Click here for additional data file. Supplemental material, sj-docx-1-wmr-10.1177_0734242X221138497 for Hydrothermal liquefaction of sewage sludge anaerobic digestate for bio-oil production: Screening the effects of temperature, residence time and KOH catalyst by Stian Hersvik Hegdahl, Camilla Løhre and Tanja Barth in Waste Management & Research
