# Supplementary material for: Hydrothermal liquefaction of sewage sludge anaerobic digestate for bio-oil production: Screening the effects of temperature, residence time and KOH catalyst
Source: Waste Manag Res. 2022 Nov 19;41(5):977–86. doi: 10.1177/0734242X221138497 (PMC10170558; doi:10.1177/0734242X221138497)
Supplement: sj-docx-1-wmr-10.1177_0734242X221138497 – Supplemental material for Hydrothermal liquefaction of sewage sludge anaerobic digestate for bio-oil production: Screening the effects of temperature, residence time and KOH catalyst [file sj-docx-1-wmr-10.1177_0734242X221138497.docx]

Supplementary Material:

**Table S1.** Elemental composition of bio-oil

| **Experiment** | **280.2.H2O** | **280.2.KOH** | **280.6.H2O** | **280.6.KOH** | **280.6.KOH_2** | **330.4.H2O** | **330.4.KOH** |
| --- | --- | --- | --- | --- | --- | --- | --- |
| $\boldsymbol{C}_{\boldsymbol{dry}} \left( \boldsymbol{wt}\boldsymbol{\%} \right)$ | 71.4 | 65.4 | 70.8 | 70.0 | 66.8 | 69.1 | 69.4 |
| $\boldsymbol{H}_{\boldsymbol{dry}} \left( \boldsymbol{wt}\boldsymbol{\%} \right)$ | 9.8 | 9.3 | 9.7 | 9.5 | 8.5 | 9.2 | 8.8 |
| $\boldsymbol{N}_{\boldsymbol{dry}} \left( \boldsymbol{wt}\boldsymbol{\%} \right)$ | 5.2 | 4.2 | 5.0 | 5.0 | 5.3 | 3.6 | 4.4 |
| $\boldsymbol{O}_{\boldsymbol{dry}} \left( \boldsymbol{wt}\boldsymbol{\%} \right)$ **^a)^** | 13.5 | 21.0 | 14.4 | 15.3 | 19.3 | 18.0 | 17.2 |
| ${\boldsymbol{H}/\boldsymbol{C}}_{\boldsymbol{molar} \boldsymbol{ratio}}$ | 1.63 | 1.69 | 1.64 | 1.62 | 1.51 | 1.59 | 1.52 |
| ${\boldsymbol{N}/\boldsymbol{C}}_{\boldsymbol{molar} \boldsymbol{ratio}}$ | 0.06 | 0.06 | 0.06 | 0.06 | 0.07 | 0.04 | 0.06 |
| ${\boldsymbol{O}/\boldsymbol{C}}_{\boldsymbol{molar} \boldsymbol{ratio}}$ | 0.14 | 0.24 | 0.15 | 0.16 | 0.22 | 0.20 | 0.19 |
| **HHV (MJ/kg) ^b)^** | 35.0 | 31.5 | 34.6 | 34.0 | 31.2 | 33.1 | 32.8 |
| **ER (%) ^c)^** | 49.7 | 59.4 | 53.9 | 54.8 | 47.4 | 76.3 | 86.2 |
|  |  |  |  |  |  |  |  |
| **Experiment** | **380.2.H_2_O** | **380.2.KOH** | **380.6.H_2_O** | **380.6.H_2_O_2** | **380.6.H_2_O_3** | **380.6.KOH** | **380.6.KOH_2** |
| $\boldsymbol{C}_{\boldsymbol{dry}} \left( \boldsymbol{wt}\boldsymbol{\%} \right)$ | 74.9 | 73.9 | 75.0 | 73.5 | 75.6 | 70.0 | 71.8 |
| $\boldsymbol{H}_{\boldsymbol{dry}} \left( \boldsymbol{wt}\boldsymbol{\%} \right)$ | 9.6 | 9.3 | 9.8 | 8.8 | 9.4 | 9.4 | 8.6 |
| $\boldsymbol{N}_{\boldsymbol{dry}} \left( \boldsymbol{wt}\boldsymbol{\%} \right)$ | 4.3 | 4.4 | 3.7 | 3.6 | 4.0 | 3.0 | 3.9 |
| $\boldsymbol{O}_{\boldsymbol{dry}} \left( \boldsymbol{wt}\boldsymbol{\%} \right)$ **^a)^** | 11.1 | 12.2 | 11.5 | 14.0 | 11.0 | 17.5 | 15.5 |
| ${\boldsymbol{H}/\boldsymbol{C}}_{\boldsymbol{molar} \boldsymbol{ratio}}$ | 1.53 | 1.50 | 1.56 | 1.43 | 1.49 | 1.60 | 1.43 |
| ${\boldsymbol{N}/\boldsymbol{C}}_{\boldsymbol{molar} \boldsymbol{ratio}}$ | 0.05 | 0.05 | 0.04 | 0.04 | 0.05 | 0.04 | 0.05 |
| ${\boldsymbol{O}/\boldsymbol{C}}_{\boldsymbol{molar} \boldsymbol{ratio}}$ | 0.11 | 0.12 | 0.12 | 0.14 | 0.11 | 0.19 | 0.16 |
| **HHV (MJ/kg) ^b)^** | 36.2 | 35.5 | 36.5 | 34.5 | 36.3 | 33.6 | 33.6 |
| **ER (%) ^c)^** | 77.6 | 79.3 | 85.0 | 81.9 | 77.2 | 94.4 | 84.8 |

^a)^ Oxygen content is found by difference. ^b)^ HHV is found by the formula presented by Channiwala et al. (Channiwala and Parikh 2002) ^c)^ Energy recovery

**Table S2.** Compounds identified in the silylated oils

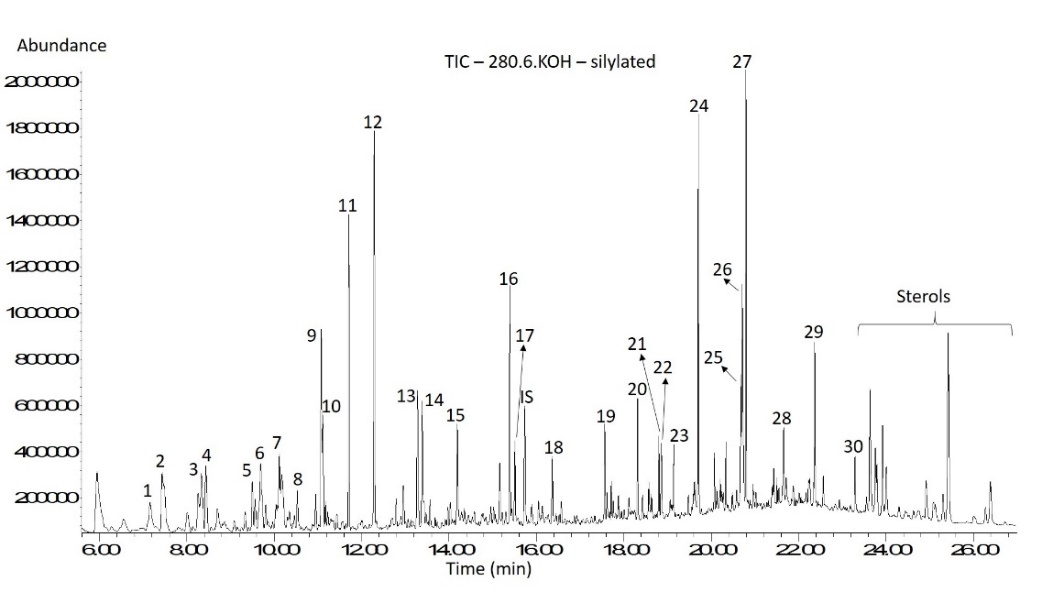

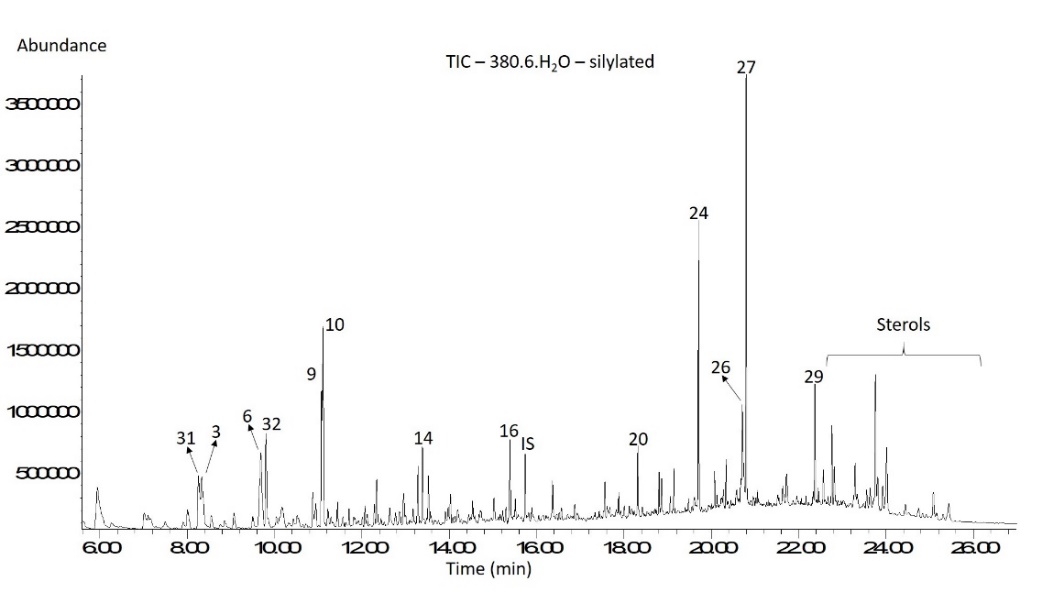


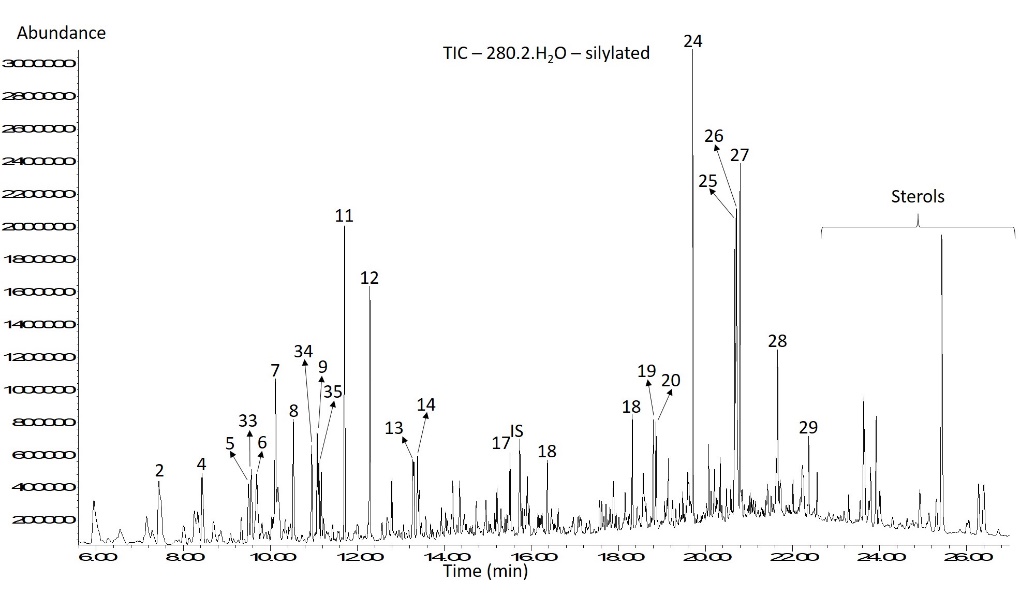


**Figure S1.** GC chromatograms of selected silylated samples. For compound identification, see Table S2. Note that for the longer aliphatic chains, there are no differentiations between straight chains and branched chains (e.g. compound **21** and **22** are both identified as C_15_ carboxylic acid, while at least one of these has to be branched).

# References

Channiwala, S. A. and P. P. Parikh (2002). "A unified correlation for estimating HHV of solid, liquid and gaseous fuels." Fuel **81**(8): 1051-1063.
